# A goal management intervention for polyarthritis patients: rationale and design of a randomized controlled trial

**DOI:** 10.1186/1471-2474-14-239

**Published:** 2013-08-13

**Authors:** Roos Y Arends, Christina Bode, Erik Taal, Mart AFJ Van de Laar

**Affiliations:** 1 Arthritis Centre Twente, Enschede, The Netherlands; 2 Department of Psychology, Health & Technology, University of Twente, P.O. Box 217, 7500 AE, Enschede, The Netherlands; 3 Department for Rheumatology, Medisch Spectrum Twente, Enschede, The Netherlands

**Keywords:** Adjustment, Self-management, Psychological well-being, Depression, Chronic disease, Adaptation, Threatened goals, Rheumatoid arthritis, Coping, Positive psychology

## Abstract

**Background:**

A health promotion intervention was developed for inflammatory arthritis patients, based on goal management. Elevated levels of depression and anxiety symptoms, which indicate maladjustment, are found in such patients. Other indicators of adaptation to chronic disease are positive affect, purpose in life and social participation. The new intervention focuses on to improving adaptation by increasing psychological and social well-being and decreasing symptoms of affective disorders. Content includes how patients can cope with activities and life goals that are threatened or have become impossible to attain due to arthritis. The four goal management strategies used are: goal maintenance, goal adjustment, goal disengagement and reengagement. Ability to use various goal management strategies, coping versatility and self-efficacy are hypothesized to mediate the intervention’s effect on primary and secondary outcomes. The primary outcome is depressive symptoms. Secondary outcomes are anxiety symptoms, positive affect, purpose in life, social participation, pain, fatigue and physical functioning. A cost-effectiveness analysis and stakeholders’ analysis are planned.

**Methods/design:**

The protocol-based psycho-educational program consists of six group-based meetings and homework assignments, led by a trained nurse. Participants are introduced to goal management strategies and learn to use these strategies to cope with threatened personal goals. Four general hospitals participate in a randomized controlled trial with one intervention group and a waiting list control condition.

**Discussion:**

The purpose of this study is to evaluate the effectiveness of a goal management intervention. The study has a holistic focus as both the absence of psychological distress and presence of well-being are assessed. In the intervention, applicable goal management competencies are learned that assist people in their choice of behaviors to sustain and enhance their quality of life.

**Trial registration:**

Nederlands Trial Register = NTR3606, registration date 11-09-2012.

## Background

The World Health Organization’s definition of health was first stated in 1948 as: ‘*a state of complete physical, mental and social well-being and not merely the absence of disease or infirmity*’ [1]. Current health systems are still, to a large extent, organized around treatment and cure of specific diseases, reflecting only the second part of the WHO’s definition of health. This results in a focus on disease instead of on health and well-being. We believe that, particularly in the case of chronic diseases, the focus needs to shift to stimulate adaptation to disease and to achieve well-being. We introduce an intervention aimed at people with a chronic condition, based on the capacities and needs of the individual person. The new intervention is based on goal management and is designed to improve peoples’ adaptation to their condition of polyarthritis. Therefore, the intervention focuses on increasing psychological and social well-being and decreasing symptoms of affective disorders. In this article, the theoretical background and the content of the intervention are described. Furthermore, we describe the design of a randomized controlled trial on the effectiveness of the intervention for increasing adaptation to polyarthritis.

### Adaptation to chronic disease

Suffering a chronic disease increases the risk for the development of secondary conditions and disabilities that often lead to further declines in health status, independence, functional status, life satisfaction, and overall quality of life [2]. Aside from the physical effects and requirements concerning lifestyle changes, a chronic disease often has major psychological and social consequences for patients. Instead of being seen as a ‘distinct biological entity existing alone and apart from the person’ [3], a chronic disease often becomes part of the identity of a person. In essence, all chronic diseases present a similar set of challenges to the patients and their families including dealing with symptoms, disability, emotional impact, complex medical regimens, difficult lifestyle adjustments, and securing helpful medical care [4]. According to the International Classification of Functioning, Disability and Health (ICF), individuals with chronic and disabling conditions are fully capable of being healthy and experiencing a satisfying subjective quality of life [2,5]. Notwithstanding this perspective, psychological distress is common in persons with polyarthritis [6], indicating that adaptation to the disease is not necessarily natural. For example, patients with rheumatoid arthritis (RA), one of the most common forms of polyarthritis, experience elevated levels of depressive mood and anxiety in comparison with healthy controls [7,8]. Research indicates 20 to 40% of RA patients suffer from heightened depression and anxiety levels [7,9-12], and depressive and anxiety symptoms are seen as key indicators of unsuccessful adaptation to polyarthritis. However, the absence of psychological distress is not the only essential outcome of adaptation; well-being is similarly essential [5,12,13]. For example, emotional well-being is found to predict long-term prognosis of physical illness; higher levels of emotional well-being tend to benefit recovery and survival rates [14].

The inclusion of the following three constructs reflects a comprehensive view on adaptation: positive affect, purpose in life and social participation. Studies have shown that, when levels of pain are elevated in patients with arthritis, positive affect was able to prevent an increase in negative affect [15,16]. Furthermore, the level of purpose in life of people with RA was found to be lower in comparison with healthy persons [17] and other studies showed that polyarthritis had a negative influence on the social participation and the work ability of the people affected [18-20]. We hope to capture the full effect of the goal management intervention by taking into account the influence the intervention has on both negative and positive indicators of adaptation to polyarthritis.

### Self-management versus health promotion

The management of most chronic diseases is an extensive responsibility that takes place mostly outside the healthcare system, as people have to manage a chronic disease everyday in combination with possibly conflicting roles and tasks [21]. In fact, the patient, family and community have become active participants in managing chronic disease [22]. Therefore, active self-management and interventions supporting patients in the acquisition of skills and techniques to that help patients learn to live with their disease are seen as essential [23]. A wide variety of self-management interventions has been developed for several chronic conditions. Self-management is ‘*the individuals’ ability to manage the symptoms, treatment, physical and psychosocial consequences and lifestyle changes inherent in living with a chronic condition*’ [24]. Reasonable evidence exists that self-management interventions are beneficial for a wide population of people with chronic diseases, for example, persons with diabetes, hypertension, heart disease and macular degeneration [25,26].

Despite receiving substantial attention in the literature, fewer benefits attained by persons with inflammatory arthritis have been reported [25]. Usually the effects of self-management interventions found for people with inflammatory arthritis are negligible to small [23,27]. Also, improvements are rarely sustained over a longer time (e.g., 9 to 14 months follow-up) [27-29]. The aforementioned term ‘self-management’ is used in literature to describe both health-oriented and disease management interventions [26], and this may cause confusion regarding the content and focus of interventions. The most frequently offered and studied self-management program for people with arthritis is the Arthritis Self-Management Course [30,31], in which common problems with day-to-day care of arthritis patients are central.

Most self-management interventions deal with the medical and behavioral management of a chronic disease, but changing roles and emotional distress due to the disease are not systematically incorporated into intervention programs [26]. Health protection or disease management interventions are motivated by the desire to control and manage illness and its consequences [32] and accommodate the unilateral focus on disease and disability. Health promotion, in contrast, is not disease or illness specific, but has illness or disability as context [33]. Moreover, health promotion interventions are intended to promote health and well-being, reflecting the aforementioned perspective of the ICF [5] that individuals with chronic and disabling conditions are fully capable of being healthy and experiencing a satisfying quality of life. Although both health promotion interventions and disease management interventions may focus on similar behaviors (e.g., exercise and medication adherence for persons with arthritis), there is a critical difference in the key outcomes assessed. Studies on disease management interventions usually do not include positive psychological and social well-being measures as outcomes. As opposed to common outcome measures of disease-specific self-management interventions (e.g., pain and disability), the outcomes of health promotion interventions should reflect the broad perspective of the WHO on adaptation to a chronic disease [5]: the experience of quality of life and being healthy in psychological and social terms.

This article introduces a health promotion intervention based on goal management theory developed for people with polyarthritis. Instead of a focus on the management of the disease (as, for example, delivered by the Arthritis Self-management Course), attention is given to how the patient can cope with activities and life goals that have become impossible to attain or are threatened due to arthritis [12]. We will explain the theory upon which the intervention is based in the next sections.

### A health promotion intervention based on goal management: a different approach

The key features of the goal management intervention arise from the characteristics of a health promotion intervention (Table 1). Although these key features are interrelated, we briefly discuss them individually. Firstly, the aim of the intervention is to improve psychological health as well as social and physical functioning. These concepts are intertwined and the intervention is, therefore, aimed to all three concepts. Secondly, the aim of the intervention follows the idea that a holistic approach comprises all aspects of the patient’s life. Thirdly, the perspective is person-focused as opposed to an orientation towards a patient-centered disease held by most self-management interventions. In a person-focused view, body systems are seen as interrelated [34] and the illness as experienced by the patient becomes central. This perspective is opposed to a disease or outsiders’ viewpoint. In a disease perspective, the focus is placed on a set of symptoms that together form a disease, which implies a particular treatment. Multimorbidity or psychosocial problems play no explicit role in this perspective. The fourth point is that the content of the goal management intervention centers around capabilities and personal potential, and, therefore, can be applied in different disease populations.

**Table 1:** Goal management intervention versus disease-specific self-management interventions

***Difference in:***	***Disease-specific self-management intervention***	***Goal management intervention***
Aim of intervention	Control and management of disease	Maximizing psychological health, social and physical functioning
Focus / approach	Reductionist	Holistic
Perspective	Patient-centered (disease-specific orientation) | Outsiders’ perspective (disease)	Person-focused (body systems are interrelated) | Insider’s perspective (illness)
Content	Disease-specific	Multiple-related diseases | Not disease-specific
Subject-matter	Predetermination of course content	Room for personal problems and difficulties
Acquisition of	Specific competencies for predetermined assumed problems	General multi-deployable competencies

Based on: Starfield, 2011 [34]; Lorig & Holman, 2003 [26]; and Stuifbergen et al., 2010 [2].

Fifth, patients give substance to their own personal trajectory, in contrast to self-management interventions with a predetermined course content [26]. The sixth distinctive feature is that patients learn general applicable goal management competencies that are not disease or problem specific, but can be used in daily life for various difficult situations. Acquiring these goal management competencies aptly complements the health promotion tradition as these competencies assist people in the choice of behaviors that sustain and enhance quality of life within the context of living with a chronic disease [2]. Also, as the focus is on using the existing possibilities and social network of a person and one’s own abilities to solve problems, the goal management intervention will promote resilience. Although the intervention described in this article is, in the first instance, developed for people with polyarthritis, due to all the intervention’s key features, it can easily be adapted for other chronic diseases or disabilities.

### Goal management

The intervention was developed based on theories of goal management. Having and striving for personal goals can give structure and meaning to life and keep a person engaged in meaningful activities [35,36]. Striving for personal goals may, however, also produce negative psychological effects when people are unable to progress to a desired goal [37,38]. Goal management strategies (possible ways to react to difficulties along the path towards a goal) are intended to minimize discrepancies between the actual situation and the goals a person has [12]. The Integrated Model of Goal Management [12] combines strategies from two different theories, namely the dual process model of assimilative and accommodative coping [39-41] and the Goal adjustment model [42]. The resulting four goal management strategies can be applied in different situations. Firstly, the strategy *goal maintenance* which implies active attempts to alter unsatisfactory life circumstances and situational constraints in accordance with personal preferences [40]. Secondly, the strategy *goal adjustment* is an approach to adjust personal goals to the personal limit of what remains possible when facing difficulties on the path to a goal [40]. Goal adjustment is the revision of self-evaluative standards and personal goals in accordance with perceived deficits and losses. Thirdly, the strategy *goal disengagement* is theorized to be one of the poles of the continuum of goal adjustment, as disengagement is an ultimate form of adjusting goals, namely, letting go of goals. Goal disengagement is applied when goals are experienced as no longer attainable [35,42]. This strategy implies the withdrawing of effort and commitment from an unattainable goal, having the benefit of releasing limited resources that can be deployed for engaging in new goals and alternative goals. Finally, the strategy *goal reengagement* implies identifying, committing to and starting to pursue new goals [42]. New goals can fill the space created by the release of unattainable goals.

### Polyarthritis

For patients with polyarthritis, maintaining one’s life goals from before disease onset is often impossible [43]. The term polyarthritis is used to indicate a variety of disorders, including rheumatoid arthritis (RA), psoriatic arthritis and ankylosing spondylitis. People with polyarthritis generally experience inflammation and swelling in joints, and despite improved medical treatment in the last decades, persisting pain, fatigue, disability, deformity, distress and reduced quality of life are daily stressors that patients have to cope with [44,45]. As a consequence, patients often face difficulties with attaining or maintaining goals in several domains of life, including work, social relationships, leisure activities and domestic tasks [46,47]. The loss of valued life activities, in particular declines in the ability to perform recreational activities and engage in social interactions, is found to be a predictor of the development of depressive symptoms [48].

### Goal management and adaptation to polyarthritis

Both striving for goals (the strategies of goal maintenance and goal reengagement), as well as accepting a given situation and the scaling down of goals (goal disengagement and goal adjustment) are of great value for adaptation. A previous study indicates that the tendency to use these strategies is associated with adaptation to arthritis [12]. Especially for people with inflammatory arthritis, who must deal with the disease’s unpredictable and fluctuating course, being able to use different approaches across situations can be beneficial [49]. An intervention based on the flexible use of goal management strategies could be promising as it can teach persons to respond to the demands of any situation in an appropriate way. The ability to use a variety of strategies across different situations is denoted by coping flexibility [50]. Despite its possible benefits for adjustment, coping flexibility is rarely studied in the context of chronic disease [51]. One study showed that an increase in coping flexibility was associated with a decreased depressed mood in patients with arthritis [52].

### Aims of the current study

For methodological reasons we have chosen one primary outcome and several secondary outcomes. Because of its comparability with other research, depressive symptoms are the main focus in this study. Firstly, depression in patients with arthritis is a well-researched and documented phenomenon [48,53-55]. The second reason is that most research on goal management in other patient groups also focused on depression as (one of the) main outcome measure(s) [41,56-58]. Our main hypothesis is that the goal management intervention leads to a significant reduction of depressive symptoms in polyarthritis patients compared to the control condition. In addition, we hypothesize a significant reduction in anxiety symptoms, and a significant improvement in positive affect, purpose in life and satisfaction with participation in patients receiving the intervention as compared to the control condition. We further explore the effect of the intervention on the disease-related outcomes of pain, fatigue and physical functioning.

Moreover, we hypothesize that the ability to use all four goal management strategies and to choose between them depending on the situation mediates the intervention’s effect on depression. Goal management competencies are also hypothesized to mediate the intervention’s effect on the secondary outcomes of anxiety, positive affect, purpose in life and satisfaction with participation. Traditionally, self-efficacy for coping with disease symptoms is found to be correlated with the effect of disease-specific self-management interventions for arthritis patients [30]. Although the goal management intervention is not explicitly designed to increase self-efficacy, we plan to study self-efficacy as an additional putative mediator on the primary and secondary outcomes. Finally, the cost-effectiveness of the intervention is analyzed in terms of medical and non-medical costs. It is conceivable that, in the long run, non-medical costs might decrease because of more realistic planning behavior and decreased absenteeism from work. Additionally, a stakeholders’ analysis of the goal management intervention is executed in order to support and promote future implementation.

## Methods and design

### Participants

The study has been approved by the medical ethics committee Twente, number NL40257.044.12. Participants are recruited via arthritis clinics in four general hospitals in The Netherlands, located in the East and Southeast areas of the country. Moreover, people from existing databases of research participants are invited to participate. Also local newspapers and contacts with patient organizations are used to reach potential participants. The process for obtaining participant informed consent is in accordance with all applicable regulatory requirements.

The research population consists of people with polyarthritis (as defined by the DBC classification system) with a psychological risk profile. The specific inclusion criteria are: 1) age of 18 years or over, 2) diagnosis of polyarthritis, and 3) score of four or higher on the depression subscale of the Hospital Anxiety and Depression Scale (HADS). People with severe distress are excluded and the treating rheumatologist is informed. Severe distress is measured as a total score on the HADS (the total of both the depression and anxiety subscales) of 22 or higher. The cut-off score is based on literature [59,60]. In addition, insufficient Dutch language skills and actual enrolment in psychotherapeutic treatment at the moment of study are exclusion criteria.

### Randomization

The participants are assigned in a 1:1 ratio to either the experimental group or the control group. Patient allocation is be done by means of blocked stratified randomisation per site in random block sizes of 2 and 4 to make sure that both conditions are equally distributed in each participating hospital. The study is open label, as it is impossible, due to the nature of the program, to blind the staff and participants involved to the condition which the patient is allocated.

### Experimental condition

The program consists of six group-based meetings with 8 to 10 participants and individual homework assignments. “Doelbewust!” is a protocol-based psychosocial educational program. *Doelbewust* is the Dutch word for “purposefully” and we have translated this program name into English as “Right On Target”. The program is led by a trained nurse. Participants are introduced to different goal management strategies and learn to use these strategies to cope in a flexible way with threatened personal goals. The goal management strategies that are covered in the program are: goal maintenance, goal adjustment, goal disengagement, and goal reengagement [39-42].

Table 2 lists the topics, goals, and applied techniques for each meeting of the program. The general structure of each meeting is as follows: a short review of the contents of the previous meeting; introducing the topics of the current meeting; elaborating the topics by group discussions and by practicing in individual, dual and group exercises; and explaining homework assignments for the next meeting. A pilot was executed to test the feasibility of the program protocol.

**Table 2:** Contents of the goal management program

**Meeting**	**Topic**	**Main goals meeting**	**Applied techniques**^**#**^
1.	Arthritis in daily life	Become aware of the influence of polyarthritis in the different domains of life.	Information (general), instruction, problem identification, behavioral information (narratives), modeling (by narratives), vicarious reinforcement (narratives), comparison (with narratives and other participants), emotional social support (by other participants), set homework tasks, prompt (email after meeting to do homework).
2.	Important personal goals	Link activities that are threatened by polyarthritis with the associated higher goals. Distinguish between lower order and higher order goals. Discuss the four goal management strategies and their pros and cons and accompanying emotions.	Information (goals, pyramid and hierarchy of goals), reframing (hierarchy of lower/higher order goals), instruction, problem identification (goal hierarchy and main goals), behavioral information (goal management strategies), record antecedents and consequences of behavior (discussion of goal management strategies), modeling (by other participants), cognitive restructuring (discussion of goal management strategies), emotional social support, set homework tasks, problem identification (homework: define threatened activity)
3.	Dealing with goals	Formulate the first threatened activity for the personal trajectory. Explore the feasibility of goal management strategies for resolving threatened activity.	Information (general), feedback (group discussion on threatened activity), social comparison (group discussion), vicarious reinforcement (group discussion), general problem solving, record antecedents and consequences of behavior (mental simulation), imagery (mental simulation), mental rehearsal (mental simulation), decision making (mental simulation) reframing (of goal management strategies by mental simulation), set homework tasks, planning (homework: write action plan)
4.	Emotions & Action plan	Design action plan for the personal trajectory. Anticipate resistance for change from self and social environment.	Feedback (group discussion on action plan), social comparison (group discussion), vicarious reinforcement (group discussion), planning (action plan), coping planning (action plan), information (emotions and resistance), modeling (by personal role model), vicarious reinforcements (role model), set homework tasks, practice behavior (goal management strategy by action plan), prompt (email after meeting to execute action plan)
5.	Alternative goal management strategy & Evaluation	Evaluate action plan and the goal management strategy used. Choose new activity for personal trajectory and practice alternative goal management strategy to solve problems with the particular activity.	Goal review (evaluation execution action plan), feedback, social comparison (group discussion), general problem solving, record antecedents and consequences of behavior (mental simulation), imagery (mental simulation), mental rehearsal (mental simulation), decision making (mental simulation) reframing (of goal management strategies by mental simulation), cognitive restructuring (of goal management strategies), planning (action plan), coping planning (action plan), set homework tasks, practice behavior (execution of action plan and goal management strategy), relapse prevention (personal warning signs)
6.	Looking back and ahead	Evaluate action plan and used goal management strategies. Consolidate learned skills and competencies. Evaluate progress during program.	Goal review (evaluation execution action plan), feedback, social comparison (group discussion), relapse prevention (personal warning signs), coping planning (personal warning signs), cognitive restructuring (of goal management strategies), planning (plan actions for future), coping planning (plan actions for future)

^#^ Note. Adapted from Michie et al., 2008 [61]; Abraham & Michie, 2008 [62]; and Vriezekolk et al., 2013 [63]. **Behavioral information**: provide information about antecedents or consequences of the behavior, or consequences between them, or behavior change techniques; **Cognitive restructuring**: changing cognitions about causes and consequences of behavior; **Comparison**: provide comparative data (cf. standard, person’s own past behavior, others’ behavior); **Coping planning**: identify and plan ways of overcoming barriers; **Decision making**: generate alternative courses of action, and pros and cons of each, and weigh them against each other; **Emotional social support**: other participants and trainer listen, provide empathy and give generalized positive feedback; **Feedback**: of (self-) monitored behavior; **Goal review**: assess extent to which the target behavior is achieved, identify factors influencing this achievement and amend target if appropriate; **Imagery**: use planned images to implement behavior change techniques; **Modeling**: observe the behavior of others; **Planning**: identify component parts of behavior and make a plan to execute each one or consider when and/or where a behavior will be performed, i.e. schedule behaviors; **Prompt**: stimulus that elicits behavior (incl. telephone calls or email reminders designed to prompt the behavior); **Record antecedents and consequences of behavior**: social and environmental situations and events, emotions, cognitions; **Relapse prevention**: identify situations that increase the likelihood of returning to a risk behavior or failing to perform a new behavior and help to plan how to avoid or manage the situation, so that new behavioral routines are maintained; **Social comparison**: provide opportunities for social comparison e.g., group learning; **Vicarious reinforcement**: observe the consequences of other’s behavior. *No definition available:***General problem solving**; **Information**; **Instruction**; **Mental rehearsal**; **Practice behavior**; **Problem identification**; **Reframing**; **Set homework tasks**.

#### Topics

**Figure 1:**
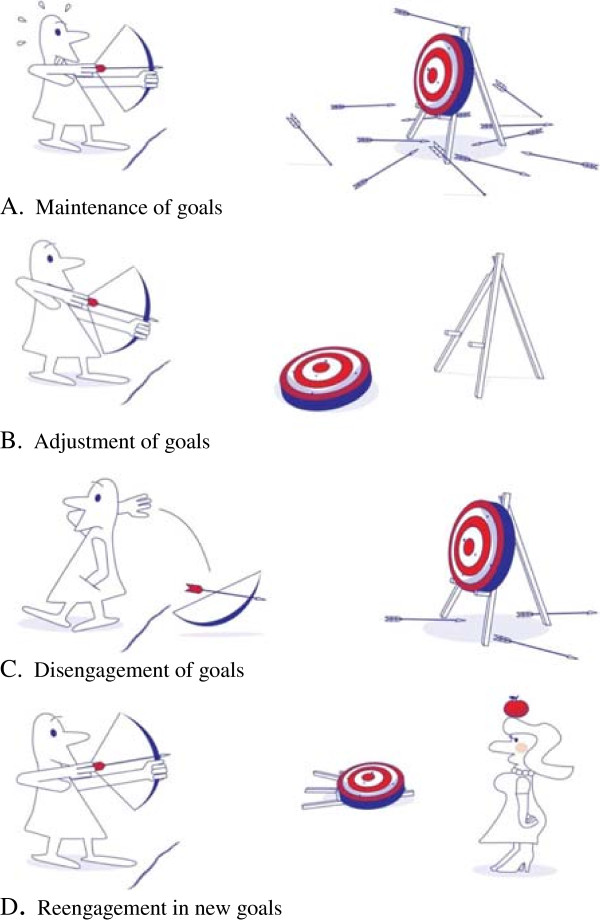
**Illustratons that depict the four goal management strategies (A-D)**^**#**^**. **^#^ Copyright: 2012 R.Y. Arends, C. Bode, E. Taal, M.A.F.J. van de Laar.

In the first meeting participants are encouraged to think about the influence arthritis has on their lives. Four narratives of fictive patients are introduced that are used throughout the whole program to discuss adaptation and the use of goal management strategies. Themes presented in the narratives are threatened personal goals, the goal management strategies, the role of the social environment, and accompanying emotions. Central in the first meeting is recognition, accomplished through comparison of the participant’s own situation with the aforementioned narratives and through the exchange of experiences with other participants. Topics in the second meeting are identifying threatened personal goals and becoming acquainted with the various goal management strategies. Participants are encouraged to explore attitudes, behaviors and emotions related to the goal management strategies, using figures that depict the various strategies (Figure 1).

**Figure 2:**
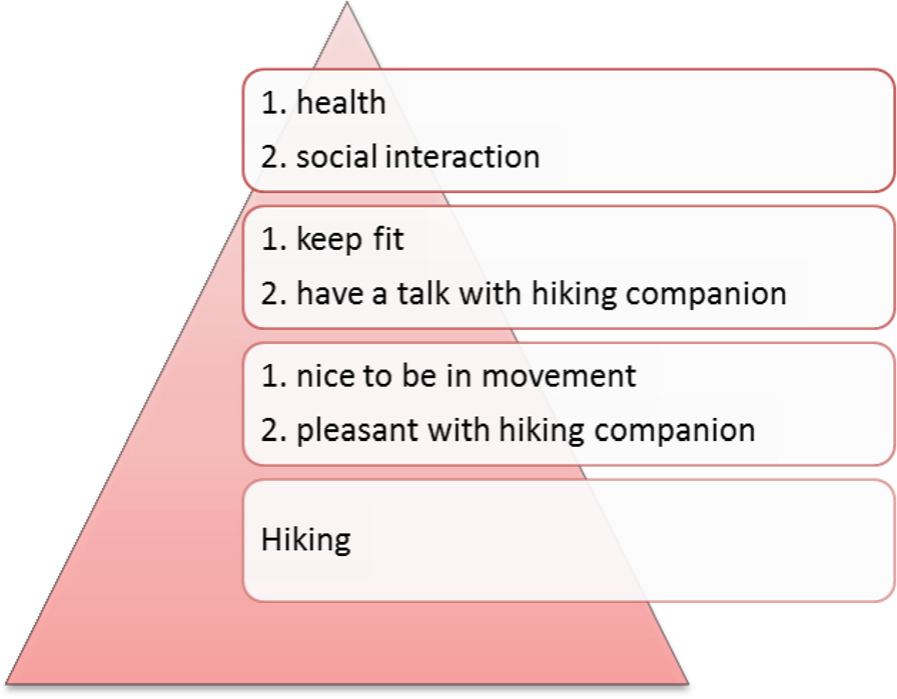
**Example of the Goal hierarchy pyramid**^**#**^**. **^#^Note. *Instructions:* Choose an activity that you care about and that is threatened by your rheumatic condition. Write the activity on the bottom layer of the pyramid. Consider whether there is a ‘higher’ goal you can put on the level above. It may help to ask yourself the following questions: Why is this important for me? What is it about the activity that appeals to me? Not all levels in the pyramid always need to be filled, just try to work your way up the pyramid as far as you can.

By formulating lower and higher order goals and discussing the goal management strategies, participants gain insights into their own behavior and preferences for strategies regarding vital threatened goals. In addition, by using the goal hierarchy pyramid (see Figure 2) to differentiate between higher order and lower order goals, participants will be helped to choose suitable goal management strategies for threatened goals at a later stage.

During the next meetings, participants choose a threatened activity and a suitable goal management strategy, and formulate and execute a personal action plan for the activity (meetings 3, 4 and 5). Every step in this process is evaluated individually by homework exercises and discussed in a group setting. In the fourth meeting, resistance to change and support from the social environment are also addressed. Central in the fifth and sixth meetings is the execution of alternative goal management strategies. In the sixth meeting, the consolidation of learned skills and competencies and the prevention of relapse in unbeneficial behavior are also addressed. Personal warning signs are used to anticipate a relapse into undesired behavior and also the use of support from the social environment is stimulated to prevent relapse.

#### Behavior change techniques

Many health psychologists have argued for a more precise description of intervention content, including specifying techniques used to accomplish behavior change [61]. The explicit communication of intervention content is necessary to study effective ingredients and to further improve the effectiveness of interventions in the future. In the context of these developments, the techniques used in the program are listed for each meeting in Table 2. A couple of techniques that originated from cognitive behavior therapy are used to stimulate the flexible use of the goal management strategies. Those techniques are used regularly in psychosocial interventions: group discussions, personal feedback, planning, self-examination and mental simulation (see Table 2 for a complete list). In particular, the technique of mental simulation is used to stimulate people to apply a new, and until now not preferred, goal management strategy. Progress to achieve goals is reached through the mental simulation of the initiation and maintenance of activities that help to reach a goal [64]. This technique has shown its feasibility and effectiveness in other studies [65,66].

#### Trainers

Specialized nurses in rheumatology care train and support the groups. Participants have the same trainer during the entire intervention period. Before the start of the program, the nurses received a full day ‘train-the-trainer’ course. In this training, the nurses worked through the entire program as a participant and completed the homework assignments to experience the techniques used in the program. In a second phase, the nurses gave the exercises themselves and received detailed feedback on their performance. At the end of the train-the-trainer course, trainers’ knowledge and skills concerning goal management and learned intervention techniques were evaluated. During the study period, trainers receive monitoring and regular supervision by a psychologist.

### Control condition

Participants in the waiting list condition do not receive the “Right On Target” program immediately. Eight months after entry into the study, which is directly after the last follow-up measurement, participants on the waiting list are invited to follow the program.

### Measurements

Table 3 gives an overview of the properties of the measurement instruments used. Participants are asked to complete six questionnaires including: intake, baseline (T0), directly after the end of the program (T1), 2 months after the end of the program (T2), 4 months after the end of the program (T3), and 6 months after the end of the program (T4). Nearly all the instruments listed in Table 3 were applied in a previous study with polyarthritis patients [12], and, when available, Cronbach’s alphas from that previous study also appear in Table 3.

**Table 3:** Study parameters, properties of the corresponding instruments and their measurement point(s)

**Content**	**Measurement point**	**Scale**	**Reference**	**Example item**	**Scale range**	**Cronbach’s alpha **^**a**^	**Items**	**Response options**
Depression	Intake, T0, T1, T4	Hospital Anxiety and Depression Scale (HADS)	Zigmond & Snaith, 1983 [60]	I have lost interest in my appearance.	0-21	.81	7	various response format (0–3)
Anxiety	Intake, T0, T1, T4	HADS	Zigmond & Snaith, 1983	I feel tense or wound up.	0-21	.83	7	various response format (0–3)
Positive affect	T0, T1, T4	Positive subscale of the Positive and Negative Affect Schedule (PANAS)	Watson, Clark, & Tellegen, 1988 [67]	Rate how you felt during the past week: e.g., *attentive*, *interested*.	10-50	.92	10	very slightly or not at all (1) - very much (5)
Purpose in life	T0, T1, T4	Purpose In Life scale (PIL)	Ryff, 1989 [68]; Ryff & Keyes, 1995 [69]	My daily activities often seem trivial and unimportant to me.	6-30	.82	6	strongly disagree (1) -strongly agree (5)
Social participation	T0, T1, T4	Family role, autonomy outdoors, social relations and work and education subscales of the Impact on Participation and Autonomy (IPA) questionnaire	Cardol, De Haan, De Jong, Van den Bos, & De Groot, 2001 [70]	Domain autonomy outdoors: The possibility to spend my (spare) time like I want to is…	0-4	.76	25	very good (0) - very poor (4)
Pain	T0, T1, T4	1 item with 100 mm visual analogue scale	-	Please indicate how much pain you had in the last 7 days due to your condition?	0-100	-	1	no pain at all (0) -unbearable pain (100)
Fatigue	T0, T1, T4	1 item with 100 mm visual analogue scale	-	Please indicate your level of fatigue averaged over the past 7 days?	0-100	-	1	no fatigue (0) -completely exhausted (100)
Physical functioning	T0, T1, T4	RAND-36 physical function subscale	Ware & Sherbourne, 1992 [71]; Van der Zee & Sanderman, 2012 [72]	Does your health limit you in these activities? If so, how much? E.g., Walking a half mile.	10-30	-	10	Yes, limited a lot (1) – No, not limited at all (3)
Goal maintenance	T0, T1, T4	Tenacious Goal Pursuit (TGP)	Brandtstädter & Renner, 1990 [73]	When faced with difficulties, I usually double my efforts.	15-75	.73	15	strongly disagree (1) -strongly agree (5)
Goal adjustment	T0, T1, T4	Flexible Goal Adjustment Scale (FGA)	Brandtstädter & Renner, 1990	I adapt quite easily to changes in plans or circumstances.	15-75	.79	15	strongly disagree (1) -strongly agree (5)
Goal disengagement	T0, T1, T4	Goal Adjustment Scale	Wrosch, Scheier, Miller, et al., 2003 [42]	If I have to stop pursuing an important goal in my life, *it’s easy for me to reduce my effort towards a goal.*	4-20	.53	4	strongly disagree (1) -strongly agree (5)
Goal reengagement	T0, T1, T4	Goal Adjustment Scale	Wrosch, Scheier, Miller, et al., 2003	If I have to stop pursuing an important goal in my life, *I seek other meaningful goals.*	6-30	.88	6	strongly disagree (1) -strongly agree (5)
Goal management strategies	T0, T1, T4	Goal Management Strategy Vignettes (GMSV)		See article’s text	-	-	-	Open ended
Coping versatility	T0, T1, T4	Coping Flexibility Questionnaire (COFLEX)	Vriezekolk, Van Lankveld, Eijsbouts, Van Helmond, Geenen, & Van den Ende, 2012 [51]	I can easily change my approach if necessary.	9-36	-	9	rarely or never (1) –almost always (4)
Self-efficacy pain	T0, T1, T4	Arthritis Self-Efficacy Scale	Lorig, et al., 1989 [30]	I am certain that I can keep arthritis pain from interfering with my sleep.	1-5	.83	5	strongly disagree (1) -strongly agree (5)
Self-efficacy for other symptoms	T0, T1, T4	Arthritis Self-Efficacy Scale	Lorig, et al., 1989	I am certain that I can control my fatigue.	1-5	.82	6	strongly disagree (1) -strongly agree (5)
Demographics	T0	Sex, age, marital status, education and current state of employment	-	-	-	N.A.	6	various response format
Disease characteristics	T0	Diagnosis and disease duration	-	-	-	N.A.	2	Various response format
Co-morbidities	T0	Checklist with 15 categories of conditions ^b^	Based on the International Classification of Diseases (ICD-10: WHO, 1992)	-	0-16	N.A.	16	-
Medication use	T0, T1, T2, T3, T4	-	-	See article’s text	N.A.	N.A.	2	See text
Utilities	T0, T1, T4	EQ-5D	Brooks, 1996 [74]; Lamers, McDonnell, Stalmeier, Krabbe, & Busschbach, 2006 [75]	I have no problems in walking about.	1-3	-	15	No problem (1) - extreme problems (3)
Direct medical costs	T0, T1, T2, T3, T4	-	-	See article’s text	N.A.	N.A.	3	Open ended
Indirect non-medical costs	T0, T1, T2, T3, T4	-	-	See article’s text	N.A.	N.A.	7	Open ended
Price estimate	T1	-	-	See article’s text	N.A.	N.A.	1	In euros for de complete course

^a^ Arends et al., 2013; ^b^ Respondents could also indicate ‘*other conditions not listed*’.

### Primary outcome measure

#### Depressive symptoms

The *depression* subscale of the Hospital Anxiety and Depression Scale (HADS) [60] is used. The HADS was chosen because the scale is designed to measure the presence and severity of depressive and anxiety symptoms whilst limiting any confounding effects of physical illness symptoms by excluding somatic items. The HADS is used both in earlier studies with arthritis patients (e.g., [54] and in studies on goal management [57]), which facilitates comparison of study results. The HADS is also used in other interventions intended to influence depressive symptoms [76]. Concurrent validity of the HADS is very satisfactory and the measure has sufficient internal consistencies [59,60].

### Secondary outcome measures

#### Anxiety

The *anxiety* subscale of the HADS [60] is used to measure anxiety symptoms.

#### Positive affect

The *positive* subscale of the Positive and Negative Affect Schedule (PANAS) [67] is used for the measurement of positive affect.

#### Purpose in life

To assess the extent wherein participants experience a meaningful life, the Purpose In Life Scale (PIL) [68,69] is used. This scale is comprised of 5 items about a person’s experience with respect to the presence or absence of life goals. One question about everyday purpose in life is added to the PIL: ‘*Doing the things I do everyday is a source of deep pleasure and satisfaction*’.

#### Participation

The Impact on Participation and Autonomy (IPA) [70] questionnaire assesses the participants’ satisfaction with social participation. We use the subscales *family role*, *autonomy outdoors*, *social relations*, and *work and education* to quantify impediments in participation and autonomy.

#### Pain

The severity of pain in the past week is measured by a 1-item visual analogue scale.

#### Fatigue

The severity of fatigue in the past week is measured by a 1-item visual analogue scale.

#### Physical functioning

The physical functioning subscale of the RAND-36 [71,72,77] is selected to measure physical functioning.

### Measures of mediation variables

To measure the use of goal management strategies, we use three generic disease questionnaires, and one disease-specific instrument especially designed to measure goal management in arthritis patients.

#### Maintenance of goals and adjustment of goals

The tendencies to use the strategies maintaining goals and adjusting goals are measured by the Tenacious Goal Pursuit and Flexible Goal Adjustment scales (FLEXTEN: TGP & FGA subscales) [73].

#### Goal disengagement and goal reengagement

The tendencies to use the strategies goal disengagement and goal reengagement are measured with the Goal Adjustment Scale (GAS) [42].

#### Goal management strategy vignettes

We have developed vignettes for explorative purposes in this program called Goal Management Strategy Vignettes (GMSV). Three vignettes are used to measure the extent in which a person is flexible in thinking about goal management. The vignettes are short stories of a person with arthritis who struggles with threatened personal goals in the domains of social relationships, leisure time and autonomy. To respond, the participant writes down possible ways in which the vignette character can react to the situation described. To analyze the answers, deductive coding for similarity with pre-defined strategies is used. To measure flexibility in thinking of goal management, the increase in the number of strategies that are mentioned per time point is used.

#### Coping versatility

The *versatility* subscale of the Coping Flexibility Questionnaire (COFLEX) [51] is used to measure coping versatility.

#### Self-efficacy

To measure self-efficacy for coping with disease symptoms, the Arthritis Self-efficacy Scale (ASES) [30,78]*pain* and *other symptoms* subscales are used.

### Demographics and disease related measures

#### Demographics

Baseline measurement includes demographic variables, including sex, age, marital status, education and current state of employment.

#### Disease characteristics

Diagnosis, disease duration (in years since diagnosis) and number of co-morbidities are asked. The diagnosis is checked by a rheumatologist.

#### Medication use and change of medication

Use of medication is asked with an open-ended question: ‘*What medications do you currently use, as prescribed by your rheumatologist?*’ Furthermore, changes in medication are asked with the question: ‘*Has anything changed in your medication in the past two months?*’ Response options are: ‘*No*’ and ‘*Yes, I started a new drug. / I stopped a drug. / The dose of a drug is increased. / The dose of a drug has been reduced’ .*

### Measures for the economic evaluation

#### Utilities

The EuroQol-5D (EQ-5D) [74,75] is used to assess utilities. The EQ-5D descriptive system consists of five dimensions: mobility, self-care, usual activities, pain/discomfort and anxiety/depression [75]. Furthermore, the ‘thermometer’ is asked; patients rate their health status on a scale from 0 (worst possible health) to 100 (best possible health).

#### Direct medical costs

Medical costs are collected on a bimonthly basis. Patients are asked the number of telephone consultations they have with their GP, as well as their number of visits to the GP, medical specialist, other paramedical and alternative therapists, and hospital days.

#### Indirect non-medical costs

Indirect non-medical costs are collected at the same frequency as the direct medical costs. Patients are asked about their absenteeism from work, domestic care, domestic help, and informal care.

#### Price estimate

Participants in the program group are asked at the post-intervention T1 measurement how many euros they would spend for participation in this program if no health insurance would pay the costs.

### Measures for the process evaluation

A brief questionnaire about the general evaluation of the session, the content, material, exercises and the presentation of the trainer is completed by the participants after each session. The trainers also fill in a short evaluation form at the end of each session and a comprehensive evaluation sheet at the end of each program.

### Measures for the competence of group trainers

During the first two courses, the trainers receive one-hour supervision after every session, and afterwards supervision occurs less frequently, but on a regular basis. Random sessions are recorded with a voice recorder and checked for correct delivery of the protocol.

### Stakeholders’ analysis measures

At the end of a program, two participants per group are randomly chosen and interviewed to evaluate the program with the use of a structured interview scheme. At the end of the study, all the trainers and one person of the management team of the participating clinics are asked during a structured interview to evaluate the program and give suggestions for implementation.

### Sample size

The sample size calculation is based on depressive symptoms as a primary objective. In order to demonstrate a medium-sized effect (Cohen’s *d* = 0.40), 100 participants in each condition are required, based on a statistical power (1-beta) of 0.80, a two-sided test and an alpha of 0.05 (power calculation with G-power).

### Analysis

#### Preliminary analysis

A flow chart of participation during the total study will be drawn. Reasons for dropout will be summarized. Percentages of missing values and dropout will be displayed. Background variables and summarized scores on questionnaires will be given. One-way ANOVA’S and *χ*^2^-tests will be performed to check for differences between the two conditions at baseline for any of the demographic variables and/or outcome measures. Intention to treat-analyzes will be conducted with use of baseline (T0) or post-intervention (T1) data depending on the last present measurement data.

#### Effectiveness analysis on primary and secondary outcome measures

To examine differences between the two conditions on all outcome measures, analysis of variance for repeated measures (group x time) will be used. If demographic or outcome measures differ significantly between the groups at baseline, these measures will be incorporated into the analysis as covariate(s). Planned polynomial contrasts are used to analyze differences in effect of the program post-intervention and after the follow-up in the experimental group. Effect sizes of the experimental group in primary and secondary outcomes at post-intervention and follow-up will be calculated with Cohen’s D using the means and pooled standard deviations of the measurements of the conditions. Effect sizes are considered according to Cohen [79] as follows: small d=0.2, medium d=0.5, and large d=0.8. To see if subgroups (e.g., high/low age, gender, disease severity) respond better to the program, subgroup analyzes will be calculated using independent t-tests or Mann–Whitney tests.

#### Analysis of mediation

Multiple mediation analysis will be performed to analyze whether the tendencies to use goal management strategies and coping versatility mediate the effects in the intervention. Primary and secondary outcomes used are the measures of depression, anxiety, purpose in life, positive affect and participation. Baseline and post-intervention measurements of both intervention and waiting list group will be used. A change score for tendencies to use goal management strategies and coping versatility will be computed with scores from baseline and post-intervention measurement. Multiple mediation analysis with bootstrapping procedures (n = 5000 bootstrap re-samples) will be used to assess the indirect effect of the mediation pattern, as recommended by Preacher and Hayes [80]. An indirect effect will be considered significant in the case when zero is not contained in the 95% bias-corrected confidence interval. Self-efficacy pain and self-efficacy for other symptoms will be incorporated in the multiple mediation analyzes as putative mediators. To analyze whether the GSMV mediate the effects of the intervention on the primary and secondary outcome measures, dummy variables will be used to indicate whether or not a strategy is named.

#### Economic evaluation

Results will be expressed as quality-adjusted life years (QALYs). The time-integrated summary score, which is the area under the curve (AUC) of the utilities, will be calculated to define the quality of life per time period (0–2 months and 0–8 months). Between-group differences in QALYs will be analyzed per period using t-test for unpaired observations. The costs will be presented as an arithmetic mean (+− SD) per patient per group. The between-group differences in resource use will be analyzed per period using the Mann–Whitney U test. For every patient and study period, the mean incremental costs will be calculated, and, using double-sided bootstrapping, 95% confidence intervals (95%CI) will be estimated. The incremental cost utility ratio (ICER) will be calculated by dividing the extra costs for the goal management intervention by the extra QALYs derived from the goal management intervention. The ICER will be expressed as costs per QALY gained. The 95% confidence intervals of the ratios will be estimated with bootstrapping. Cost evaluations will be conducted from the societal perspective and with a time-horizon of less than one year. Due to this short time-horizon, costs and effects will not be discounted.

## Discussion

The purpose of the presented study protocol is to evaluate the effectiveness of the “Right On Target” program, a newly developed goal management intervention. We predict that the experimental condition will show positive effects compared with usual care in reducing depressive and anxiety symptoms and in improving positive affect, purpose in life and social participation, and will be cost effective. Both the program itself and its evaluation are likely to add to the existing body of knowledge in several ways, as described below.

### Strengths and limitations of the goal management intervention

To the best of our knowledge “Right On Target” is the first program that focuses on the four goal management strategies of goal maintenance, goal adjustment, goal disengagement and goal reengagement to support improvement of adaptation to a chronic disease. The possibility to tailor the program to the personal needs of participants is expected to increase its effectiveness and participants’ commitment to the program.

We provided a detailed description of the ingredients of the intervention, in accordance with the argument of Michie et al. [61], in order to communicate applied techniques that support the development of effective interventions and to improve knowledge regarding effective behavior change techniques. We hope to be able to identify the active ingredients in our intervention by clearly stating the underlying theory and assumed mechanisms of behavioral change.

Furthermore, the present study incorporates a holistic focus on adaptation, as the outcomes assessed are both the absence of psychological distress and the presence of well-being. Hence, this research focuses not only on the difficulties that people may experience due to a chronic disease, but also on personal sources of resilience.

As stated earlier in this paper, the goal management program focuses on dealing with threatened personal goals, rather than a pre-defined focus on disease-related goals. This program may ask for different competencies than health professionals are used to deploying in their daily practice. The specialized nurses in rheumatology care who provide the program have undergone extensive training. During the study period, the nurses receive regular guidance and supervision. Nevertheless, it is possible that nurses find it difficult to “sit on their hands” and not provide immediate solutions. This program might be less suitable without the extensive training and guidance of the nurses.

### Strengths and limitations of the randomized controlled trial

Our study will hopefully answer questions regarding the effectiveness of the goal management intervention for patients with a rheumatic disease. We try to understand the pathways that are responsible for successful adaptation in persons dealing with a rheumatic disease and investigate who benefits most. In addition, we have included an economic evaluation. However, these additional analyzes cannot be conducted in absence of an effect of the goal management intervention compared to the waiting list group.

As the “Right On Target” program is a newly developed intervention, we execute a stakeholders’ analysis. Experiences of participants, trainers and the management of the participating clinics provides insight into the feasibility regarding the intervention. The information from the stakeholders’ analysis supports future implementation of the intervention.

## Conclusion

To test the effectiveness of the “Right On Target” program to increase adaptation to polyarthritis, a randomized controlled trial is needed and a design for this study is presented. Results from this trial will test the effectiveness of the “Right On Target” program in improving the adaptation of patients to polyarthritis in terms of the absence of psychological distress and the presence of well-being. The protocol for the randomized controlled trial reflects a comprehensive view both on adaptation and on goal management. The presented study will add to the existing body of knowledge of health promotion interventions.

## Competing interests

The authors declare that they have no competing interests.

## Authors’ contributions

RYA, CB, ET and MAFJL were responsible for conceiving and designing the study. All authors contributed to the development and critical evaluation of the study protocol. RYA drafted the manuscript and the other authors revised it critically and corrected draft versions. All authors read and approved the final manuscript.

## Pre-publication history

The pre-publication history for this paper can be accessed here:

http://www.biomedcentral.com/1471-2474/14/239/prepub
